# MiR-215-5p is a tumor suppressor in colorectal cancer targeting EGFR ligand epiregulin and its transcriptional inducer HOXB9

**DOI:** 10.1038/s41389-017-0006-6

**Published:** 2017-12-04

**Authors:** Petra Vychytilova-Faltejskova, Jana Merhautova, Tana Machackova, Irene Gutierrez-Garcia, José Garcia-Solano, Lenka Radova, Dominika Brchnelova, Katerina Slaba, Marek Svoboda, Jana Halamkova, Regina Demlova, Igor Kiss, Rostislav Vyzula, Pablo Conesa-Zamora, Ondrej Slaby

**Affiliations:** 1 Central European Institute of Technology, Masaryk University, Brno, Czech Republic; 2 Department of Comprehensive Cancer Care, Masaryk Memorial Cancer Institute, Faculty of Medicine, Masaryk University, Brno, Czech Republic; 3 Department of Pharmacology, Faculty of Medicine, Masaryk University, Brno, Czech Republic; 4 Department of Clinical Analysis, Santa Lucia University Hospital, Cartagena, Spain; 5 Department of Pathology, Santa Lucia University Hospital, Cartagena, Spain

## Abstract

Growing evidence suggests that microRNAs are involved in the development and progression of colorectal cancer (CRC). In the present study, deregulation and functioning of tumor-suppressive miR-215-5p was evaluated in CRC. In total, 448 tumor tissues and 325 paired adjacent healthy tissues collected from Czech and Spain cohorts of CRC patients have been used for miR-215-5p expression analyses. A series of in vitro experiments have been performed using transient transfection of miR-215-5p mimics into four CRC cell lines to identify specific cellular processes affected by miR-215-5p. Further, the effects of miR-215-5p on tumor growth were evaluated in vivo using NSG mice and stable cell line overexpressing miR-215-5p. Target mRNAs of miR-215-5p were tested using luciferase assay and western blot analyses. We found that miR-215-5p is significantly downregulated in tumor tissues compared with non-tumor adjacent tissues and its decreased levels correlate with the presence of lymph node metastases, tumor stage, and shorter overall survival in CRC patients. Overexpression of miR-215-5p significantly reduced proliferation, clonogenicity, and migration of CRC cells, lead to cell cycle arrest in G2/M phase and p53-dependent induction of apoptosis. The ability of miR-215-5p to inhibit tumor growth was confirmed in vivo. Finally, we confirmed epiregulin and HOXB9 to be the direct targets of miR-215-5p. As epiregulin is EGFR ligand and HOXB9 is its transcriptional inducer, we suggest that the main molecular link between miR-215-5p and CRC cells phenotypes presents the EGFR signaling pathway, which is one of the canonical pathogenic pathways in CRC.

## Introduction

Colorectal cancer (CRC) is the third most common cancer worldwide and the fourth leading cause of cancer related deaths. Despite the fact that the incidence and mortality rates have been steadily declining, >50% of all patients with CRC will die of the disease^1^. In recent years, many different classes of non-coding RNAs have been identified as key regulators of various cellular processes including cell proliferation, differentiation, apoptosis or migration^2–5^. MicroRNAs (miRNAs) are short single-stranded non-coding RNAs that post-transcriptionally regulate gene expression by binding to 3′ untranslated regions of target mRNAs^6^. Many studies have shown they can act as both oncogenes and tumor suppressors and their deregulation has been associated with the initiation and progression of a wide range of human diseases, including cancer^7, 8^. In addition, association between miRNA expression, prognosis and therapy response prediction was repeatedly described^9, 10^.

Over the past decade, several miRNAs with deregulated expression in CRC have been identified, including miR-215-5p^11–15^. We focus on miR-215-5p as we identified this miRNA to be downregulated in colorectal tumor tissue in our previous work^11^, where it indicated also promising tumor-suppressive features in preliminary *in vitro* functional screen^11^. In general, this miRNA is supposed to function as a tumor suppressor and its levels are often downregulated in tumor tissues. However, its role in CRC pathogenesis has not been fully elucidated yet. In 2008, miR-215 has been shown to act as an effector as well as regulator of p53^13^. Further, denticleless protein homolog^14^ and thymidylate synthase^15^ were confirmed to be the miR-215-5p targets. Low expression levels of miR-215-5p were associated with resistance to 5-fluorouracil-containing adjuvant chemotherapy^16^. Finally, the deregulation of this miRNA is supposed to be a very early event, which is not dependent on the mechanism of initiation of transformation, suggesting that miR-215-5p is likely to regulate critical signaling pathways that are crucial for early transformation of colonic epithelial cells^12^.

In this study, we have determined expression levels of miR-215-5p in two large independent cohorts of CRC patients to confirm its downregulation in tumor tissue and prognostic potential. To further discover the role of miR-215-5p in CRC pathogenesis, we have performed deep *in vitro* analyses with the aim to describe the most significantly affected CRC cells phenotypes and identify mRNA targets and the key signaling pathways affected by miR-215-5p. The role of miR-215-5p in regulation of tumor growth was evaluated also *in vivo* using mouse model.

## Results

### MiR-215-5p is downregulated in CRC tissues and its low levels correlate with aggressive disease

**Fig. 1:**
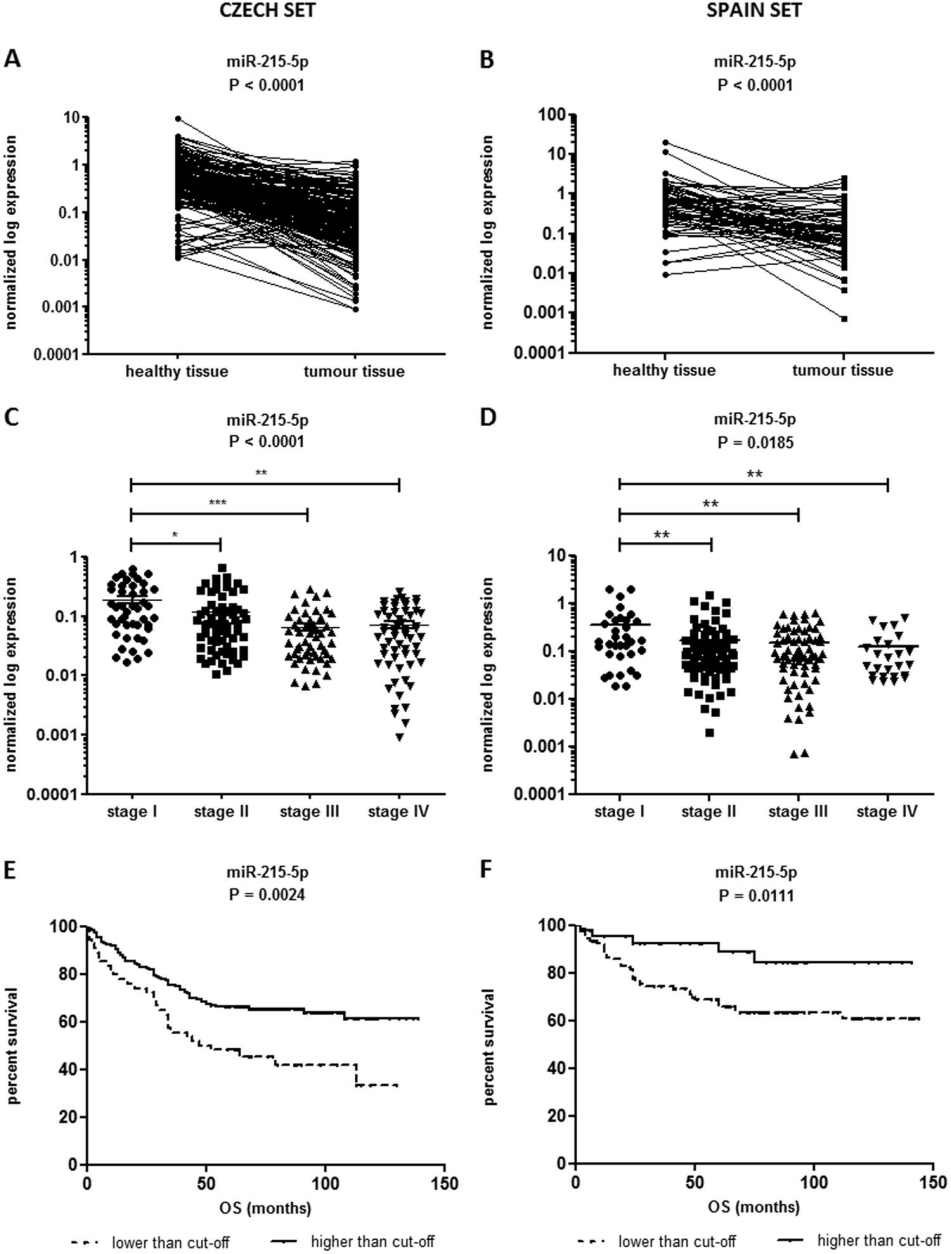
Expression analyses of miR-215-5p in CRC patients **a** Expression levels of miR-215-5p are significantly decreased in CRC tissues compared with healthy adjacent tissues (*P* < 0.0001; Czech cohort). **b** Expression levels of miR-215-5p are significantly decreased in CRC tissues compared with healthy adjacent tissues (*P* < 0.0001; Spain cohort). **c** Expression of miR-215-5p significantly decreases with advanced clinical stage (*P* < 0.0001; Czech cohort). **d** Expression of miR-215-5p significantly decreases with advanced clinical stage (*P* = 0.0185; Spain cohort). **e** Low levels of miR-215-5p correlate with shorter OS of Czech CRC patients (*P* = 0.0024). **f** Low levels of miR-215-5p correlate with shorter OS of Spain CRC patients (*P* = 0.0111). *OS* overall survival

It was confirmed that the expression of miR-215-5p is significantly downregulated in tumor tissue compared with adjacent mucosa (*P* < 0.0001; Fig. 1a) in case of Czech cohort (Table 1). In addition, the levels of miR-215-5p decreased progressively with advanced clinical stages (*P* < 0.0001; Fig. 1c) and low expression was associated with lymph nodes positivity (*P < *0.0001; Supplementary Fig. S1A). Further, significantly downregulated levels of miR-215-5p were found not only in primary tumors, but also in corresponding liver metastases (*P* < 0.0001; Supplementary Fig. S1B). Survival analyses proved that patients with low levels of miR-215-5p have significantly shorter overall survival (OS) (*P* = 0.0024; cut-off 0.02393; Fig. 1e) compared with patients with higher expression levels.

**Table 1:** Correlation of miR-215-5p expression with clinical-pathological features of CRC patients

	*n* (%)	miR-215-5p Czech cohort median (25–75%)	*n* (%)	miR-215-5p Spain cohort median (25–75%)
Age				
Median (range)	66 (18–92)	NA	73 (36–91)	NA
Sex				
Male	139 (55)	0.07 (0.02–0.15)	107 (55)	0.07 (0.03–0.17)
Female	113 (45)	0.07 (0.03–0.014)	89 (45)	0.10 (0.04–0.20)
*P*-value		0.9673		0.3508
Tumor vs. mucosa				
Normal mucosa	252	0.51 (0.33–0.87)	73	0.58 (0.27–1.19)
Colorectal tumor	252	0.07 (0.03–0.14)	196	0.11 (0.05–0.25)
*P*-value		**<0.0001**		**<0.0001**
Clinical stage	*n = *252		*n* = 192	
I	43 (17)	0.14 (0.07–0.31)	23 (12)	0.15 (0.09–0.43)
II	78 (31)	0.07 (0.03–0.13)	82 (43)	0.08 (0.05–0.16)
III	60 (24)	0.04 (0.02–0.08)	64 (33)	0.08 (0.04–0.20)
IV	71 (28)	0.05 (0.02–0.12)	23 (12)	0.05 (0.03–0.15)
*P*-value		**<0.0001**		**0.0185**
pT category	*n* = 252		*n* = 193	
pT1	4 (2)	0.30 (0.08–0.60)	10 (5)	0.04 (0.03–0.08)
pT2	51 (20)	0.09 (0.05–0.26)	18 (9)	0.05 (0.01–0.20)
pT3	172 (68)	0.06 (0.02–0.13)	124 (65)	0.09 (0.04–0.16)
pT4	25 (10)	0.04 (0.02–0.09)	41 (21)	0.10 (0.05–0.20)
*P*-value		**0.0018**		0.2515
Lymph nodes	*n = *252		*n* = 192	
Negative	135 (54)	0.09 (0.04–0.21)	110 (57)	0.09 (0.05–0.19)
Positive	117 (46)	0.04 (0.02–0.10)	82 (43)	0.08 (0.04–0.20)
*P*-value		<**0.0001**		0.4397
Distant metastases	*n = *252		*n* = 193	
No	181 (72)	0.07 (0.03–0.14)	170 (88)	0.08 (0.04–0.15)
Yes	71 (28)	0.06 (0.02–0.15)	23 (12)	0.14 (0.03–0.30)
*P*-value		0.2780		0.3306
Grading	*n = *252		*n* = 196	
G1	67 (27)	0.07 (0.03–0.26)	144 (73)	0.09 (0.04–0.20)
G2	132 (52)	0.07 (0.03–0.12)	47 (24)	0.08 (0.04–0.14)
G3	53 (21)	0.04 (0.02–0.16)	5 (3)	0.13 (0.02–0.31)
*P*-value		0.1771		0.4259
Tumor location	*n = *252		*n* = 194	
Proximal colon	100 (40)	0.07 (0.03–0.18)	104 (54)	0.09 (0.04–0.19)
Distal colon	152 (60)	0.07 (0.02–0.13)	90 (46)	0.07 (0.03–0.17)
*P*-value		0.4787		0.1817

The *P*-values in bold are statistically significant

*NA* not applicable

To further validate these observations, an independent cohort from Spain was included in the study (Table 1). As in the Czech cohort, the expression of miR-215-5p was significantly downregulated in tumor tissues (*P* < 0.0001; Fig. 1b) and its low levels were associated with advanced clinical stage (*P* = 0.0185; Fig. 1d), but not with the lymph node positivity (Table 1). Again, the low levels of miR-215-5p were associated with shorter OS and worse prognosis of CRC patients (*P* = 0.0111; cut-off 0.2139; Fig. 1f).

### MiR-215-5p expression levels in CRC cells

MiR-215-5p expression levels in CRC cells used in our study was performed by use of calibration curve (Supplementary Fig. S7A) and absolute quantification. The number of miR-215-5p copies varied among CRC cells (Supplementary Fig. S7B). HCT-116^+/+^, HCT-116^−/−^, DLD-1 and HT-29 were characteristic with very low number of miR-215-5p copies ranging from 1351 to 3639 copies per 100 ng of total RNA purified from CRC cells. On the contrary, the only CRC cells indicating multiple time higher levels were CaCo2 cells with number of copies 98 962 per 100 ng of total RNA. Based on this results, HCT-116^+/+^, HCT-116^−/−^, DLD-1 and HT-29 cells were used as models for miR-215-5p substitution and CaCo2 for miR-215-5p silencing.

### MiR-215-5p inhibits proliferation, viability and colony formation of CRC cells

**Fig. 2:**
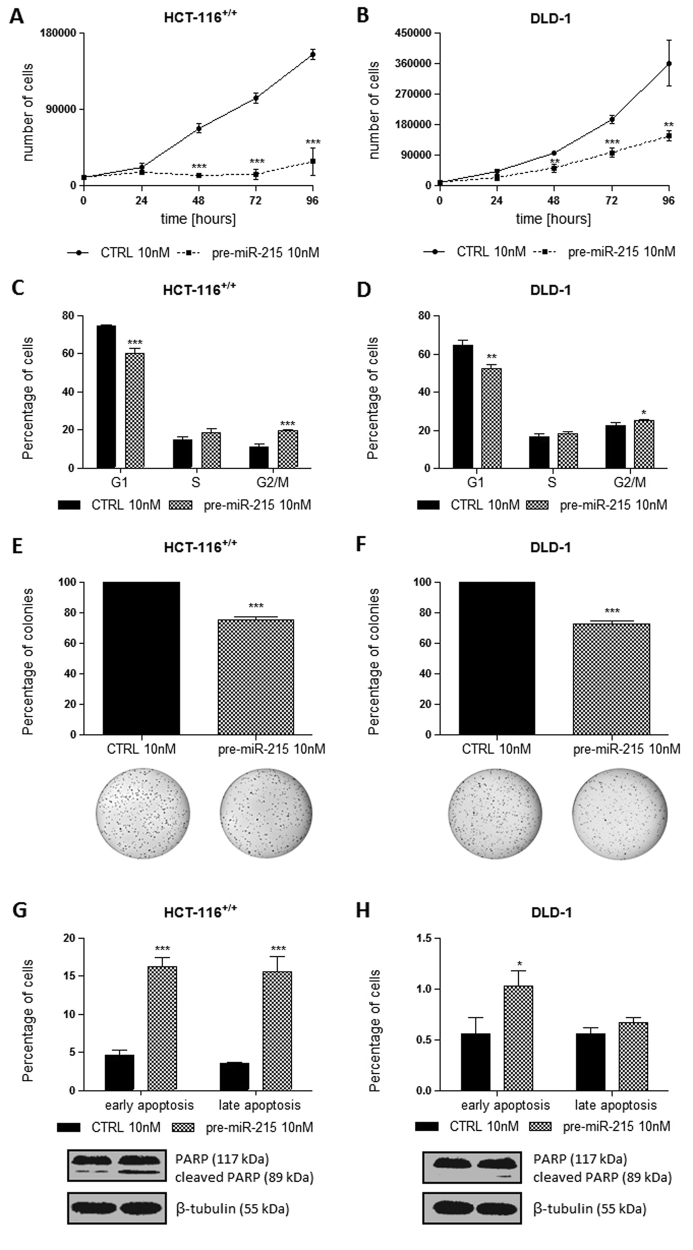
Effects of miR-215-5p overexpression on HCT-116^+/+^ and DLD-1 cells **a** miR-215-5p significantly inhibits the proliferation of HCT-116^+/+^ cells. **b** miR-215-5p significantly inhibits the proliferation of DLD-1 cells. **c** miR-215-5p significantly reduce the clonogenicity of HCT-116^+/+^ cells. **d** miR-215-5p significantly reduce the clonogenicity of DLD-1 cells. **e** Overexpression of miR-215-5p in HCT-116^+/+^ cells leads to a cell cycle arrest in G2/M phase. **f** Overexpression of miR-215-5p in DLD-1 cells leads to a cell cycle arrest in G2/M phase. **g** miR-215-5p increases the apoptosis of HCT-116^+/+^ cells in p53-dependent manner. **h** miR-215-5p increases the early apoptosis of DLD-1 cells. *P < 0.05, **P < 0.01, ***P < 0.001, CTRL control cells

By transfection of miR-215-5p, mimic reached significant increase of miR-215-5p levels in all studied cell lines, which was stable from 24 to 96 h. Expression levels of miR-215-5p in cells transfected with miR-215-5p mimic were 8000–10 000 times higher when compared with mock-transfected control cells. Cell counting demonstrated cell proliferation to be inhibited by ectopic expression of miR-215-5p, with the best inhibition effect being observed 96 h after transfection (*P* < 0.001 for HCT-116^+/+^ and HT-29; *P* < 0.01 for DLD-1 and HCT-116^−/−^; Figs. 2a, b, Supplementary Figs. S2A, B). Parallel to cell counting, MTT (3-(4,5-dimetylthiazol-2-yl)-2,5-difenyltetrazolium bromid) assay was performed to assess the effect of miR-215-5p on cell viability. Similarly to the previous results, the viability of CRC cells was significantly reduced 96 h after transfection (*P* < 0.001 for HCT-116^+/+^ and HT-29; *P* < 0.01 for DLD-1; *P* < 0.05 for HCT-116^−/−^). To determine whether the alterations in cell proliferation and viability were the result of cell cycle regulation, flow cytometry was used. Ninety-six hours after transfection, miR-215-5p decreased the proportion of HCT-116^+/+^ (Fig. 2c), HCT-116^−/−^ (Supplementary Fig. S2C) and DLD-1 (Fig. 2d) cells in the G0/G1-phase and increased the proportion of the cells in S-phase and G2/M-phase compared with those transfected with control oligonucleotides. In case of HT-29 cells, only the arrest in G2/M-phase was observed (Supplementary Fig. S2D). To find out whether the inhibition of growth induced by miR-215-5p was anchorage independent, the cells were seeded on soft agar 24 h post-transfection. After 14 days, HCT-116^+/+^ and DLD-1 cells transfected with miR-215-5p mimics formed significantly fewer colonies than cells transfected with control oligonucleotide (*P* < 0.001; Figs. 2e, f).

### MiR-215-5p induces apoptosis of CRC cells in a wild-type p53-dependent manner

Transfection of miR-215-5p into HCT-116^+/+^ cells (wt-p53) significantly increased the number of apoptotic cells by at least threefold (*P* < 0.001; Fig. 2g). Further, the levels of cleaved PARP were increased subsequent to transfection with miR-215-5p (Fig. 2g). Importantly, transfection of miR-215-5p mimics into HCT-116^−/−^ (p53-null), DLD-1 (mut-p53) and HT-29 (mut-p53) cells did not lead to increased apoptosis rates and elevated levels of cleaved PARP (Fig. 2h, Supplementary Figs. S2E–F).

### MiR-215-5p inhibits migration of CRC cells

**Fig. 3:**
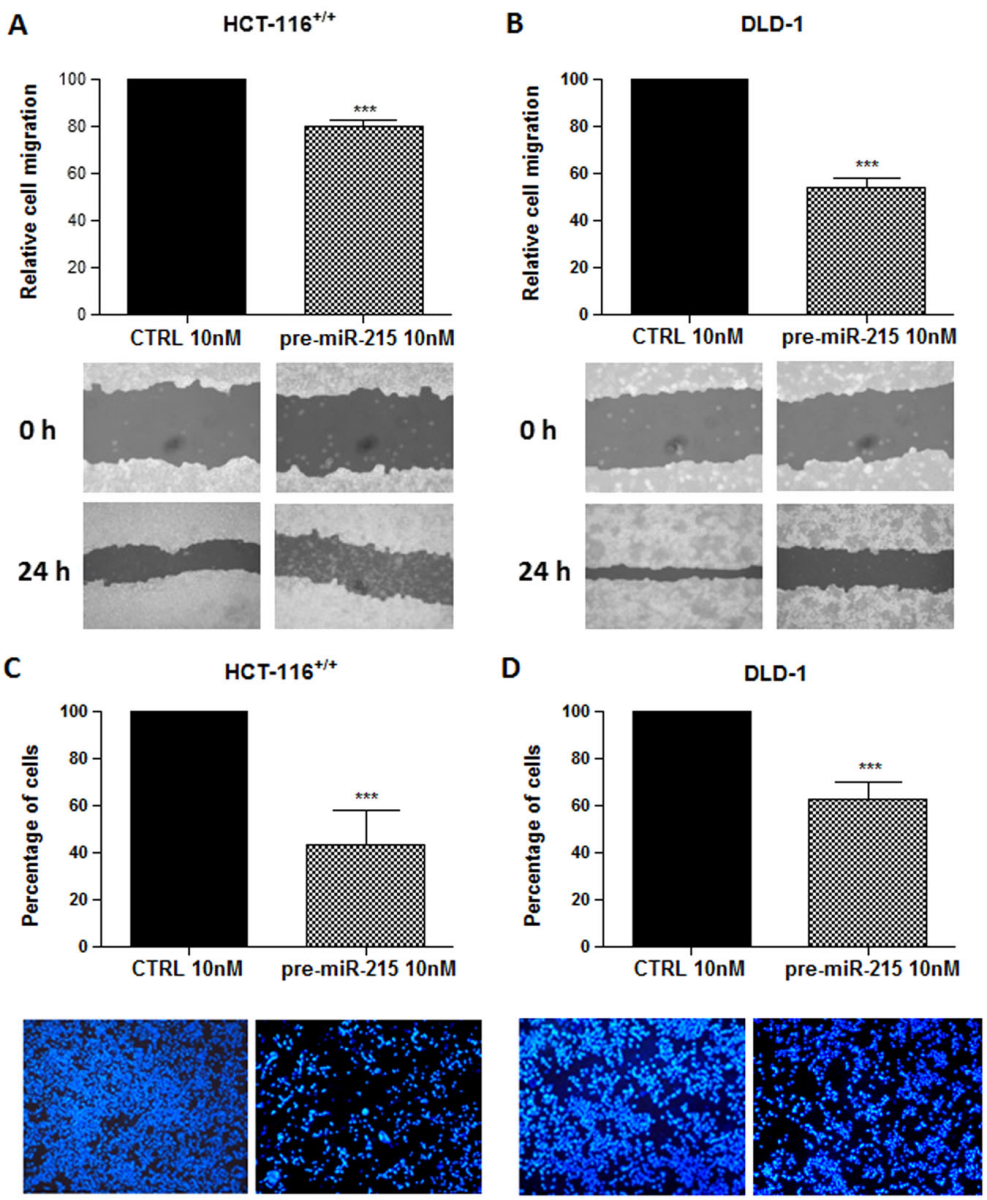
Effects of miR-215-5p overexpression on migration of HCT-116^+/+^ and DLD-1 cells **a** miR-215-5p significantly reduce the migration of HCT-116^+/+^ cells (transwell migration assay). **b** miR-215-5p significantly reduce the migration of DLD-1 cells (transwell migration assay). **c** miR-215-5p significantly reduce the migration of HCT-116^+/+^ cells (scratch wound assay). **d** miR-215-5p significantly reduce the migration of DLD-1 cells (scratch wound assay). **P < 0.01, ***P < 0.001, CTRL control cells

According to the results of scratch wound assay, transfection of miR-215-5p mimics led to a significant inhibition of cell migration (*P* < 0.001 for DLD-1, HCT-116^+/+^ and HCT-116^−/−^; *P* < 0.05 for HT-29; Figs. 3a, b and Supplementary Figs. S3A, B). In addition, transwell migration assay confirmed significantly reduced migration of cells overexpressing miR-215-5p. The inhibition rate was 57 ± 16%, 37 ± 8%, and 50 ± 17%, respectively, in HCT-116^+/+^ (Fig. 3c), DLD-1 (Fig. 3d), and HCT-116^−/−^ cells transfected with miR-215-5p mimics compared with control group.

### EREG and HOXB9 are direct targets of miR-215-5p

**Fig. 4:**
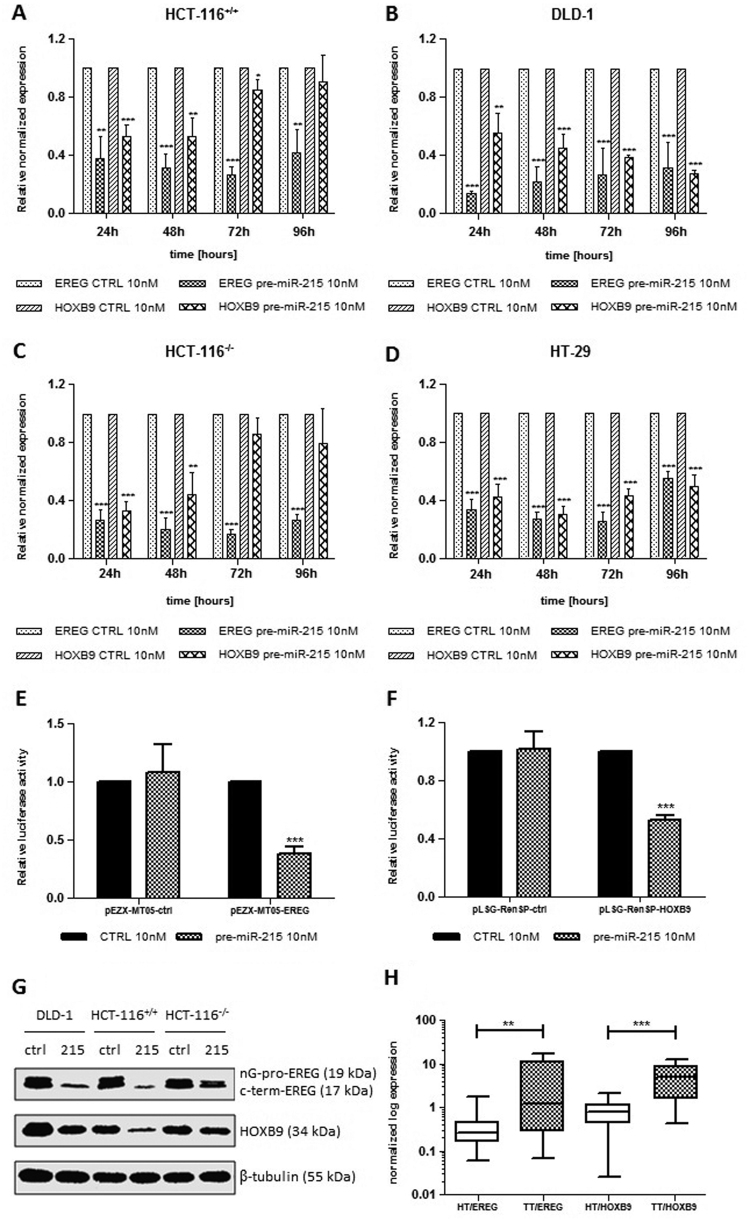
EREG and HOXB9 are direct targets of miR-215-5p **a** RT-qPCR analyses proved significantly reduced mRNA levels of EREG and HOXB9 in HCT-116^+/+^ cells. **b** RT-qPCR analyses proved significantly reduced mRNA levels of EREG and HOXB9 in DLD-1 cells. **c** RT-qPCR analyses proved significantly reduced mRNA levels of EREG and HOXB9 in HCT-116^−/−^ cells. **d** RT-qPCR analyses proved significantly reduced mRNA levels of EREG and HOXB9 in HT-29 cells. **e** Luciferase assay confirmed EREG to be a direct target of miR-215-5p. **f** Luciferase assay confirmed HOXB9 to be a direct target of miR-215-5p. **g** Western blot analyses proved downregulated protein levels of EREG and HOXB9 in CRC cells transfected with miR-215-5p mimics. **h** Expression of EREG and HOXB9 is significantly upregulated in tumor tissues compared with non-tumor adjacent tissues. *P < 0.05, **P < 0.01, ***P < 0.001, *CTRL* control cells, *HT* healthy tissue, *TT* tumor tissue

To better understand the role of miR-215-5p in CRC pathogenesis, the TargetScan^17^, DIANA-microT^18^, RNAhybrid^19^, miRanda^20^ and RNA22^21^ databases were searched for the predicted targets of miR-215-5p associated with cell proliferation and migration. Among the target genes that were considered to be the most likely involved in these processes, epiregulin (EREG)^22^ and HOXB9^23^ have been chosen for further analyses. It was found that miR-215-5p transfection leads to the significant decrease in mRNA levels of both genes of interest (Figs. 4a-d). Subsequently, the luciferase reporter assay was utilized to confirm direct interaction between miR-215-5p and 3′-UTR of EREG and HOXB9. It was shown that miR-215-5p suppressed 62 ± 6% of reporter activity of the pEZX-MT05-EREG reporter compared with the control oligonucleotide, whereas the pEZX-MT05-ctrl vector was resistant to the inhibition (*P* < 0.001; Fig. 4e). Similarly, the reporter activity of the pLSG-RenSP-HOXB9 reporter was suppressed by 48 ± 3% after transfection of miR-215-5p compared with control cells, whereas the pLSG-RenSP-ctrl vector was resistant to the inhibition (*P* < 0.001; Fig. 4f). In addition, western blot analyses proved that overexpression of miR-215-5p suppresses the expression of both proteins 48 h after transfection (Fig. 4g). To further support these data, depletion of EREG and HOXB9 using small interfering RNA (siRNA)-mediated knockdown was performed (Supplementary Figs. S4A–D). It was shown that downregulation of these two proteins leads to the significant decrease in proliferation of HCT-116^+/+^ (*P* < 0.001 in case of EREG, *P* < 0.01 in case of HOXB9; Supplementary Fig. S5A), HCT-116^−/−^ (*P* < 0.01 in case of EREG; Supplementary Fig. S5B) and DLD-1 (*P* < 0.01 in case of EREG, *P* < 0.05 in case of HOXB9; Supplementary Fig. S5C) cells 96 h after transfection. By use of scratch wound assay, we were not able to prove any significant effects of EREG and HOXB9 silencing on migratory capacity of studied cells (*P* > 0.05). Finally, the levels of EREG and HOXB9 were examined in the matched tumor and non-tumor tissues of CRC patients. It was shown that the expression of EREG and HOXB9 is significantly increased in tumor tissues compared with healthy tissues (*P* < 0.01 for EREG, *P* < 0.001 for HOXB9; Fig. 4h).

### MiR-215 induces increase in E-cadherin expression

When we compared the expression levels of EMT markers (E-cadherin, vimentin, ZEB1, ZEB2) in HCT-116^+/+^-miR-215-5p cells and HCT-116^+/+^-control cells, we observed significantly higher levels of E-cadherin (*P* = 0.0164, Supplementary Fig. S6) in miR-215-5p-positive cells. There was no difference in vimentin and ZEB1 expression levels between studied cell lines. ZEB2 was not detectable in both cell lines.

### MiR-215-5p silencing facilitate proliferation of CRC cells and induce expression of EREG and HOXB9

We successfully silenced miR-215-5p expression in CaCo2 cells to 16% of its expression levels in control CaCo2 cells transfected with anti-miRNA control oligonucleotide (Supplementary Fig. S8A). Silencing of miR-215-5p by use of anti-miR-215 in CaCo2 cells led to the increase in expression levels of miR-215-5p targets EREG and HOXB9 after 48 h (Supplementary Fig. S8B). Finally, decreased levels of miR-215-5p facilitated proliferation of CaCo2 cells, which was significant at first (*P* = 0.03) and second day (*P* = 0.02) post-transfection (Supplementary Fig. S8C). We have not observed any significant effects of miR-215-5p silencing on migratory capacity of CaCo2 cells by use scratch wound-healing assay (*P* > 0.05).

### MiR-215-5p overexpression suppresses tumor growth *in vivo*

**Fig. 5:**
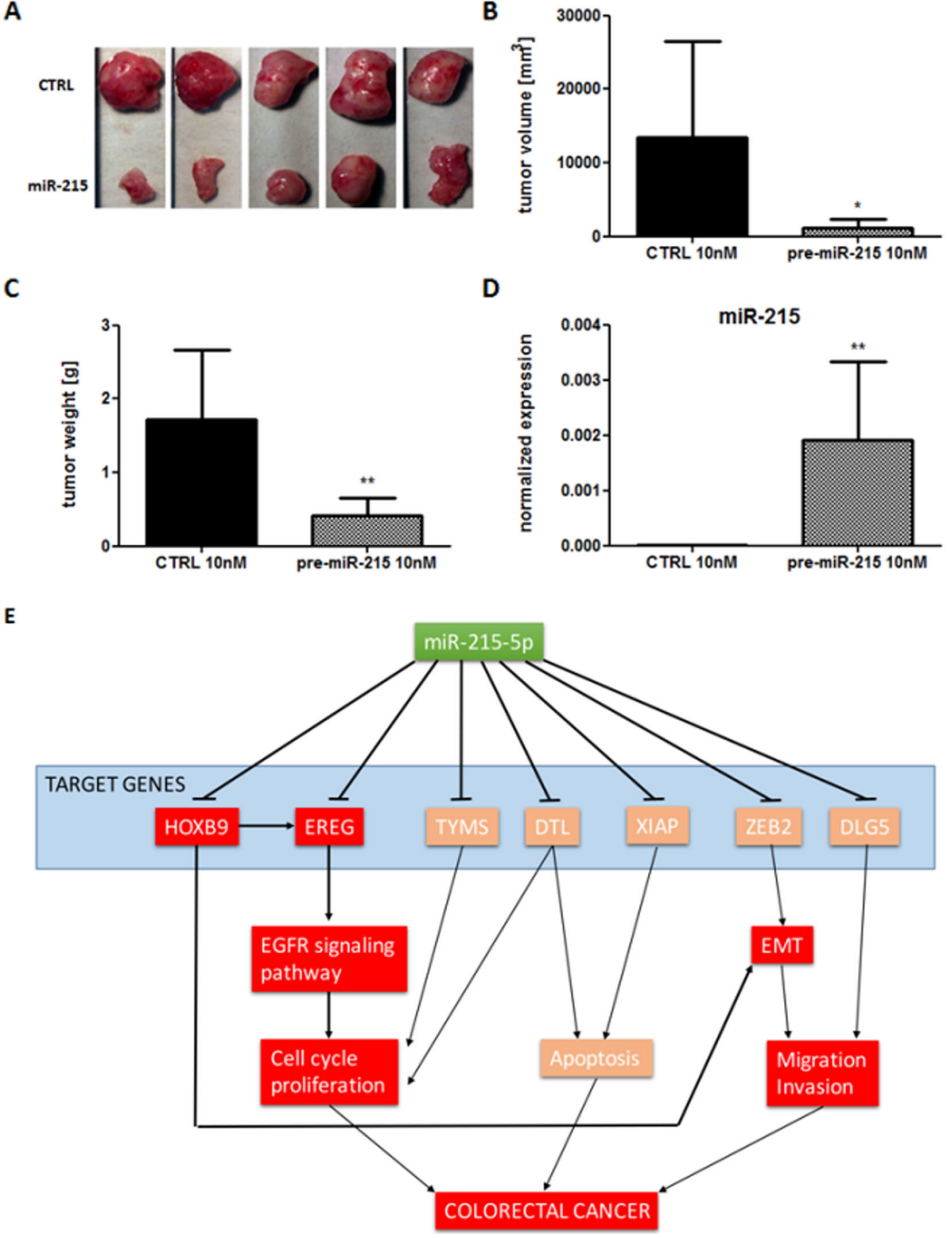
Effects of miR-215-5p overexpression on tumor growth *in vivo* and its involvement in CRC pathogenesis **a** Subcutaneously injected HCT-116^+/+^-miR-215-5p cells formed significantly smaller tumors compared with HCT-116^+/+^-control cells 25 days after application into NSG mice (*n* = 5). **b** Volume of *in vivo* formed tumors was significantly smaller in case of HCT-116^+/+^-miR-215-5p cells compared with HCT-116^+/+^-control cells. **c** Weight of *in vivo* formed tumors was significantly smaller in case of HCT-116^+/+^-miR-215-5p cells compared with HCT-116^+/+^-control cells. **d** Expression levels of miR-215-5p were significantly increased in HCT-116^+/+^-miR-215-5p tumors compared with HCT-116^+/+^-control tumors 25 days after initiation of the experiment. **e** Involvement of miR-215-5p in CRC pathogenesis—direct targets of miR-215-5p described first in this study are in red squares, direct targets of miR-215-5p described in previous studies are in brown. *P < 0.05, **P < 0.01, *CTRL* control cells, *EMT* epithelial–mesenchymal transition, *XIAP* X-chromosome-linked inhibitor of apoptosis, *ZEB2* zinc-finger E-box-binding homeobox 2, *DTL* denticleless protein homolog, *DLG5* discs large homolog 5, *TYMS* thymidylate synthase, *HOXB9* homeobox protein HoxB9, *EREG* epiregulin

To evaluate how miR-215-5p overexpression affects tumor growth *in vivo*, subcutaneous tumors were generated in NSG mice using HCT-116^+/+^-miR-215-5p cells and HCT-116^+/+^-control cells. It was confirmed that HCT-116^+/+^-control tumors grow significantly faster than the HCT-116^+/+^-miR-215-5p tumors (Figs. 5a–c). Importantly, reverse transcriptase-quantitative PCR (RT-qPCR) analysis showed that the expression of miR-215-5p is still upregulated in HCT-116^+/+^-miR-215-5p tumors compared with control tumors (Fig. 5d) 25 days from the beginning of the experiment.

## Discussion

Growing evidence suggests that miRNAs are involved in the development and progression of different types of human cancers^24–26^. In 2008, miR-215-5p was first described as a tumor suppressor in CRC^13^. Since then, several other authors studied prognostic and predictive value of miR-215-5p^14–16^; however, its detail functioning in the pathogenesis of the disease has not been clarified yet. Thus, we have analyzed not only the diagnostic and prognostic potential of miR-215-5p, but we also aimed to identify its target genes and describe its involvement in CRC cells phenotypes and particular signaling pathways.

We have confirmed that the expression of miR-215-5p is significantly downregulated in tumor tissues compared with paired healthy tissues and its reduced levels correlate with higher clinical stage, presence of lymph node metastases and shorter OS. These results are in agreement with former studies^27–30^.

To identify specific cellular processes influenced by miR-215-5p, series of in vitro experiments have been performed. The results proved that higher levels of miR-215-5p significantly reduce metabolic activity and proliferation of CRC cell lines. The opposite effects were observed when miR-215-5p silencing approach was used in CaCo2 cells, where decreased levels of miR-215-5p led to enhanced cellular proliferation. Similarly, the in vivo experiments confirmed significantly slower growth of tumors stably expressing this miRNA. In compliance with previous studies^13, 14^, the inhibitory effect was more profound in cells containing wild-type p53 (HCT-116^+/+^) compared with p53-mutant (DLD-1, HT-29) or p53-null (HCT-116^−/−^) cells. On the other hand, miR-215-5p significantly reduced cell proliferation even in the absence of p53; thus it seems that this miRNA slows down the proliferation not only through the cell cycle arrest, but also by affecting another signaling pathways independent of p53 function. Concerning the cell cycle, transfection of miR-215-5p lead to the significant arrest in G2/M phase. These results again support the hypothesis of other proteins than p53 being involved in reduced proliferation. Georges et al. confirmed denticleless protein homolog to be a direct target of miR-215-5p that interacts with DDB1-CUL4 and MDM2-p53 ligase complexes and influences the stability of p53 and its target p21^31, 32^. Similarly, Boni et al. identified thymidylate synthase as another target of miR-215-5p that was suggested to be a predictive biomarker for 5-FU response in CRC. To find out whether the inhibition of growth induced by miR-215-5p was anchorage independent, the clonogenic assay was performed^15^. It was revealed that number of colonies is significantly lower in case of HCT-116^+/+^ and DLD-1 cells transfected with miR-215-5p mimics, but not in HCT-116^−/−^ and HT-29 cells indicating that this miRNA could affect the cell clone formation by mechanisms that are at least in part dependent on p53 functionality. Interestingly, although carrying out the above experiments significant morphologic changes in HCT-116^+/+^ cells transfected with miR-215-5p, such as round shape and plate surface detachment, have been repeatedly observed. These changes may have many reasons including reduced expression of adhesion molecules, loss of cell polarity, dysfunctional cytoskeleton or cell apoptosis^33^. Georges et al. identified discs large homolog 5 as an important target of miR-215-5p^31^. It was shown that this protein can interfere with cell adhesion through the reduction of cadherin transport to the cell surface and it is proposed to function in the maintenance of epithelial cell structure^34^. Concerning the effect of miR-215-5p on cell apoptosis, it was assessed that overexpression of this miRNA leads to the significant increase of apoptotic rate in case of HCT-116^+/+^ cells. Although the exact mechanism of action is not known, it was proved that this outcome is strongly dependent on the presence of wt-p53 and could be related to morphologic changes described earlier. To date, no target genes of miR-215-5p associated with cell apoptosis have been identified in CRC. Nevertheless, X-chromosome-linked inhibitor of apoptosis (XIAP) was found to be regulated by this miRNA in ovarian^35^ and non-small cell lung cancer^36^.

Further, we observed that higher levels of miR-215-5p lead to a significant inhibition of cell migration. Interestingly, the highest effect was determined in case of DLD-1 cell line indicating the independence of p53 status. In 2011, White et al. identified ZEB2 as a direct target of miR-215-5p in renal cell carcinoma^37^. These results were further confirmed using non-small cell lung cancer^38^ and pancreatic cancer^39^ cell lines. Using the metastatic gene profiling assay, several other genes involved in the degradation of extracellular matrix or cell adhesion, such as MMP7/13 or CDH1/6/11, have been described to be affected by increased miR-215-5p expression in renal cell carcinoma^37^; however, these targets need to be further validated in CRC.

As our observations proved significant effects of miR-215-5p on cell proliferation and migration, several databases have been searched for the potential targets of miR-215-5p associated with these processes. From the predicted genes, EREG and HOXB9 have been chosen for further validation. The performed analyses proved these two proteins to be the direct targets of miR-215-5p. Moreover, their expression was significantly upregulated in tumor tissue compared with adjacent healthy tissue. EREG is a member of the epidermal growth factor family that functions as a ligand of EGFR, which is commonly overexpressed in CRC and present one of its main molecular features^38^. HOXB9 is an important transcription factor contributing to solid tumor invasion and metastasis and its overexpression is associated with poor prognosis^40, 41^. Interestingly, it was found that EREG promoter contains the HOX-binding site and is a direct transcriptional target of HOXB9^42^. To date, no previous study has confirmed EREG and HOXB9 to be direct targets of miR-215-5p. However, Wu et al. identified HOXB9 as a direct target of miR-192, a miRNA from the same family and with a high homology to miR-215-5p^43^. The most prominent regulatory effect of miR-215-1p on HOXB9 was observed under p53-wild-type conditions, in cell line HCT-116^+/+^, which could be partly explained by the fact that miR-192^13, 44^ and miR-215^44^ have been shown to be p53-responsive miRNAs. As miR-215-5p has an ability to regulate EGFR ligand EREG and its transcriptional inducer HOXB9, we suggest that the main molecular link between miR-215-5p and CRC cells phenotypes presents the EGFR signaling pathway, which is one of the canonical pathogenic pathways in CRC (Fig. 5e).

In conclusion, we have confirmed a diagnostic and prognostic potential of miR-215-5p in CRC patients in two independent cohorts of patients. In addition, we have proved the tumor-suppressive character of miR-215-5p resulting in reduced proliferation, formation of new colonies, and migration and increased apoptosis. These results correspond with the fact that one miRNA has the ability to regulate several target genes involved in different signaling pathways. Although some of these effects were dependent on the presence of wt-p53, miR-215-5p was also able to slow down the tumor growth independently of this protein. Importantly, two genes—EREG and HOXB9—that are functionally linked to EGFR signaling and are known to be involved in cell proliferation, migration and disease progression have been validated as direct targets of this miRNA. Thus, we believe that miR-215-5p could serve as a potential therapeutic target in CRC.

## Materials and methods

### Patients and tissue samples

In total, 252 tumor tissue samples from patients with histopathologically verified CRC who had undergone surgery at Masaryk Memorial Cancer Institute (Brno, Czech Republic) from 2004 to 2013, as well as 252 paired adjacent non-tumor tissues were used for the determination of miR-215-5p expression levels. In addition, 17 samples of corresponding liver metastases obtained from patients with metastatic CRC were used in our study. Further, an independent set of tumor tissues from 196 patients who had undergone the surgery at Santa Lucía General University Hospital (HGUSL, Cartagena, Spain) from 2004 to 2015, as well as 73 paired adjacent non-tumor tissues were involved in the study. All subjects enrolled in the study were of the same ethnicity (European descent) and did not receive any treatment prior to surgery. All patients were followed-up for tumor recurrence at regular intervals and survival time was calculated. Clinical and pathological characteristics were recorded and are summarized in Table 1. Written informed consent was obtained from all participants and the study has been approved by the local Ethical Boards in Masaryk Memorial Cancer Institute and Santa Lucía General University Hospital.

### Tissue samples preparation and miRNA isolation

Tissue samples were homogenized (MM301, Retsch GmbH & Co. KG, Germany) and total RNA enriched for small RNAs was isolated using *mir*Vana miRNA Isolation Kit (Ambion, Austin, TX, USA) according to the manufacturer’s instructions. Concentration and purity of RNA were determined spectrophotometrically by measuring its optical density (A260/280 > 2.0; A260/230 > 1.8) using a Nanodrop ND-1000 (Thermo Fisher Scientific, Waltham, MA, USA).

### Reverse transcription and RT-qPCR

For miRNA expression analyses, complementary DNA (cDNA) was synthesized from 10 ng of total RNA using gene-specific primers (has-miR-215-5p; ID 000518, RNU48; ID 001006) according to the TaqMan MicroRNA Assay protocol (Applied Biosystems, Foster City, CA, USA) and real-time PCR was performed using TaqMan Universal PCR Master Mix, NoUmpErase UNG (Applied Biosystems) as described previously^11^. For quantification of the number of miR-215-5p copies in CRC cells used in our study, a dilution series of synthetic miRNA oligo (IDT, Coralville, IA, USA) were carried out in parallel with qRT-PCR of biological samples to generate an absolute standard curve. MiR-215-5p levels in CRC cells were expressed as number of copies per 100 ng of total RNA purified from CRC cells. For the purposes of gene expression analyses, cDNA was synthesized using 1000 ng of total RNA and the High-Capacity cDNA Reverse Transcription Kit (Applied Biosystems) according to the manufacturer’s recommendations. Quantitative PCR was carried out using specific probes for EREG (Hs00914313_m1), HOXB9 (Hs00256886_m1), PMM1 (Hs00160195_m1), CDH1 (Hs01023895_m1), VIM (Hs00958111_m1), ZEB1 (Hs01566408_m1), ZEB2 (Hs00207691_m1) and GAPDH (glyceraldehyde-3-phosphate dehydrogenase, Hs02758991_g1) (Applied Biosystems). Real-time PCR was performed using the Applied Biosystems 7500 Sequence Detection System.

### Cell lines and cell culture

In this study, four human colon carcinoma cell lines were used including HCT-116^+/+^ (CCL-247^TM^; wt-p53), DLD-1 (CCL-221^TM^; mut-p53), HT-29 (HTB-38^TM^; mut-p53), CaCo2 (HTB-37^TM^, mut-p53) and HCT-116^−/−^ (p53-null derivative). The first four cell lines were obtained from American Type Culture Collection (ATCC), the HCT-116^−/−^ cells were kindly provided by Dr Jiri Kohoutek (Veterinary research institute, Brno, Czech Republic) who gained them from Dr Bert Vogelstein^45^. Cells were cultured in Dulbecco’s modified Eagle’s medium (DMEM) supplemented with 10% fetal bovine serum, 100 µg ml^−1^ penicilin, 100 µg ml^−1^ streptomycin, 0.1 mM non-essential amino acids, 2 mM l-glutamin, 1 mM sodium pyruvate (Invitrogen, Gibco, Carlsbad, CA, USA) in 5% CO_2_ at 37 °C. All cell lines were regularly tested with MycoAlert (Lonza Group Ltd, Basel, Switzerland) to ensure the absence of mycoplasma contamination. Authentication of cell lines was done by comparing STR (short tandem repeat) sequences obtained from actual cell lines as determined by Generi Biotech (Hradec Kralove, Czech Republic) with data public available (ATCC, ECACC—European Collection of Authenticated Cell Cultures). Recent STR analysis has been performed within 6 months before the beginning or in the course of the experiments for all cell lines.

### Cell transfection

All cell lines were transfected with 10 nM hsa-miR-215-5p mimic (MC10874) or miRNA Mimic, negative control #1 (4464058) or 33 nM hsa-miR-215-5p inhibitor (MH10874) or 33 nM miRNA inhibitor, negative control (4464079) or 30 nM siRNA-negative control (AM4635), siEREG (145900) and siHOXB9 (109525; all from Ambion) 24 h after seeding using Lipofectamine RNAiMAX transfection reagent (Invitrogen) according to the manufacturer’s protocol. Transfection efficiency was evaluated by RT-qPCR.

### Cell proliferation and MTT assay

Cells were seeded in triplicates in 10% DMEM without antibiotics in 24-well plates 24 h before transfection and counted 24–96 h after transfection. Cell viability was measured using the MTT assay (Sigma Aldrich, Saint Louis, MO, USA). The absorbance was measured on Multi-Detection Microplate Reader (BIO-TEK, Winooski, VT, USA).

### Colony-forming assay

Colony-forming assay was performed using six-well plates pre-coated with 0.75% agarose as the bottom layer, whereas the top layer consisted of 0.35% agarose and tumor cells transfected with miR-215 mimics or control oligonucleotide. After 12–14 days, colonies were stained with crystal violet blue solution (Sigma-Aldrich) and scanned by GelCount (Oxford Optronix, Abingdon, UK). The data were analyzed using ImageJ software (Wayne Rasband, NIH, MD, USA).

### Cell cycle analysis and detection of apoptosis

Cell cycle analysis and detection of apoptosis were performed using flow cytometry as described previously^11^. The cells were analyzed 72 and 96 h post-transfection.

### Scratch wound migration assay

The migration of cells was analyzed using scratch wound migration assay. Cells were seeded on six-well plates and the cell monolayer was wounded 24 h after the transfection. The migration was measured at time 0 and 24 h post-wounding using a microscope Nikon Diaphod 300 INV (10 × ) and camera Canon Power shot A95. Images were analyzed by the Tscratch software (CSElab, ETH Zurich, Switzerland).

### Transwell migration assay

Transwell migration assay was performed using 8 µm transwell inserts for 24-well plates (Costar, Corning Incorporated, Corning, NY, USA) and staining with Hoechst 33342 (Invitrogen). The migrated cells were counted using fluorescence microscope and ImageJ software (Wayne Rasband).

### Luciferase assay

For luciferase reporter assay, MISSION 3′-UTR Lenti GoClone HOXB9 (HUTR10238) and appropriate negative control (HUTR001C) from SwitchGear Genomics (Carlsbad, CA, USA) were used and the viral particles were added at MOI (multiplicity of infection) = 2.5. In case of EREG, 1 µg of pEZX-MT05 vector containing UTR for EREG (HmiT004978) or appropriate control vector (CmiT000001-MT05) were transfected into DLD-1 cells using EndoFectin Plus Transfection Reagent (GeneCopoeia, Rockville, MD, USA). The luciferase activity was measured using the MISSION LightSwitch Luciferase Assay Reagent (Sigma Aldrich) or SecretePair Dual Luminescence assay kit (GeneCopoeia), respectively, using FLUOstar Omega Microplate reader (BMG Labtech, Ortenberg, Germany).

### Western blotting

Cells were seeded in 60 mm plates and 48 h after transfection they were lysed with RIPA buffer (Sigma-Aldrich) containing Complete mini protease and phosphatase inhibitor cocktail tablets (Roche, Basel, Switzerland). Protein quantification was performed using the Bradford protein assay (Bio-Rad, Hercules, CA, USA) and 10 µg of lysate was loaded per lane. Proteins were resolved by 8 or 10% sodium dodecyl sulfate–polyacrylamide gel electrophoresis gel and wet transferred to polyvinylidene difluoride membrane (EMD Millipore, Billerica, MA, USA). The signals were visualized by ECL Prime Western blotting Detection Reagent (Amersham, Piscataway, NJ, USA) and exposed to AGFA Curix X-ray film (AGFA, Mortsel, Belgium). The following Ab were used: anti-HOXB9 (1:100, mouse, sc-398500) from Santa Cruz Biotechnology Inc. (Dallas, TX, USA) and anti-EREG (1:1000, rabbit, 12048 S), anti-PARP (1:1000, rabbit, 9542 S) and anti-β-tubulin (1:2000, rabbit, 2146 S) from Cell Signaling Technology (Danvers, MA, USA).

### Generation of stable cell line overexpressing miR-215-5p

Stable transfectants were generated using OriGene’s pCMV6-Mir vectors with miR-215-5p precursor or control sequence and TurboFectin 8.0 (OriGene Technologies, Rockville, MD, USA). Stable clones were selected using 300 µg ml^−1^ G418 (Sigma Aldrich). The stable expression of miR-215-5p was evaluated by RT-qPCR.

### In vivo tumorigenicity assay

Five NSG mice (males, 8‒10 weeks old, 21‒26 g, initially obtained from The Jackson Laboratory, Bar Harbor, USA) were housed and monitored in individually ventilated cage system (Techniplast, Buguggiate, Italy) with ad libitum access to water and feeding. The assay was performed according to the protocol described previously^46,47^. Tumors have been palpable since day 14 and mice were sacrificed on day 25. During the experiment, tumor growth and animal behavior were individually monitored. Animal experiments were performed in accordance with national and EU animal welfare legislation and all procedures were approved by institutional (Masaryk University, Brno) and national ethics committees.

### Data normalization and statistical analyses

The threshold cycle data were calculated by QuantStudio 12 K Flex software using the default threshold settings. All real-time PCR reactions were run in triplicates and average threshold cycle and SD values were calculated. The average expression levels of miR-215-5p in tumor and adjacent non-tumor tissues, as well as in the cell lines were normalized using RNU48 as a reference gene, the expression of EREG and HOXB9 was normalized using PMM1 (in case of tissue samples) or GAPDH (in case of cell lines) as a reference genes; subsequently, all data were transformed by the 2^−ΔCt^ method. Statistical differences between the levels of miR-215-5p in tumor and non-tumor tissues were evaluated by the non-parametric Wilcoxon test for paired samples. Furthermore, Mann–Whitney *U*-test was used to analyze the correlation between miR-215-5p expression levels and clinical–pathological features of the patients. Survival analyses were performed using the log-rank test and Kaplan–Meier plots approach. For *in vitro* and *in vivo* analyses, the two-sided Student's *t-*test was used to compare the mean values between two groups. Data are presented as the mean values with SD unless otherwise noted (all in vitro measurements were repeated three times). All calculations were performed using GraphPad Prism version 5.00 (GraphPad Software, San Diego, CA, USA). *P*-values of <0.05 were considered statistically significant.

## Electronic supplementary material

Supplementary Material

Supplementary Figure S1

Supplementary Figure S2

Supplementary Figure S3

Supplementary Figure S4

Supplementary Figure S5

Supplementary Figure S6

Supplementary Figure S7

Supplementary Figure S8
