# Supplementary material for: MiR-215-5p is a tumor suppressor in colorectal cancer targeting EGFR ligand epiregulin and its transcriptional inducer HOXB9
Source: Oncogenesis. 2017 Dec 4;6(11):399. doi: 10.1038/s41389-017-0006-6 (PMC5868056; doi:10.1038/s41389-017-0006-6)
Supplement: Supplementary file 1 — Supplementary Material [file 41389_2017_6_MOESM1_ESM.docx]

**SUPPLEMENTARY FIGURES**

**
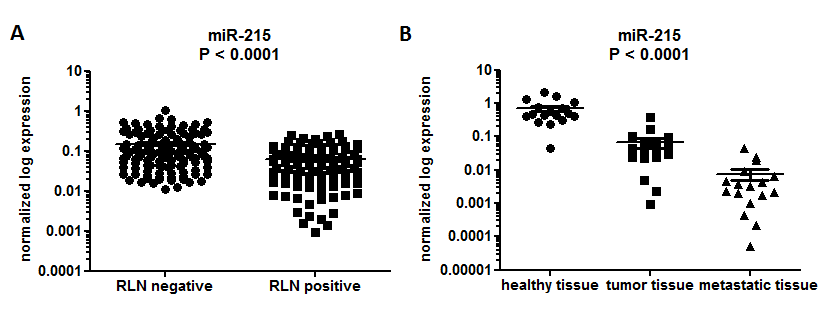
**

**Supplementary Figure S1 The expression analysis of miR-215-5p in tumour and metastatic tissue of CRC patients.** A, MiR-215-5p is down-regulated in CRC patients with lymph node metastases (*P* < 0.0001; Czech set). B, Expression of miR-215-5p is significantly decreased in both, primary tumours and liver metastases (*P* < 0.0001; Czech set). RLN – regional lymph nodes


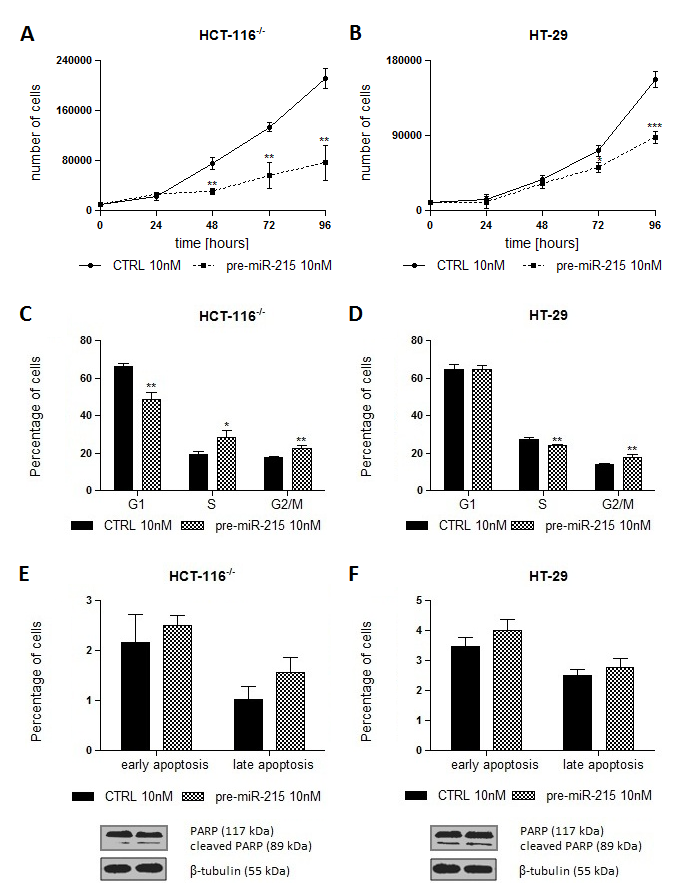


**Supplementary Figure S2 Effects of miR-215-5p over-expression on cell proliferation, cell cycle and apoptosis of HCT-116^-/-^ and HT-29 cells.** A, miR-215-5p significantly inhibits the proliferation of HCT-116^-/-^ cells. B, miR-215-5p significantly inhibits the proliferation of HT-29 cells. C, Over-expression of miR-215-5p in HCT-116^-/-^ cells leads to a cell cycle arrest in G2/M phase. D, Over-expression of miR-215-5p in HT-29 cell leads to a cell cycle arrest in G2/M phase. E, miR‑215‑5p does not increase the apoptosis of HCT-116^-/-^ cells (p53-null). F, miR-215-5p does not increase the apoptosis of HT-29 cells (mut-p53). * *P* < 0.05, ** *P* < 0.01, *** *P* < 0.001, CTRL = control cells

**
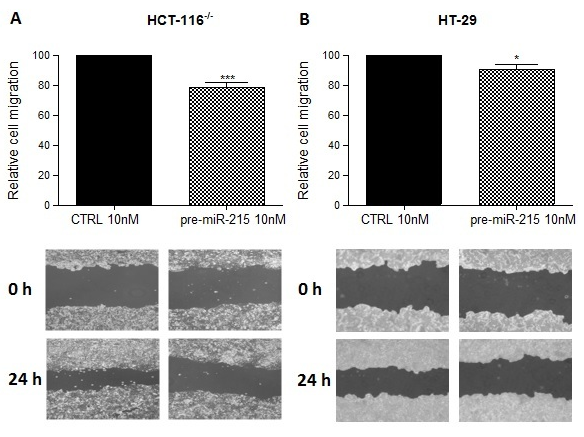
**

**Supplementary Figure S3 Effects of miR-215-5p over-expression on migration of HCT‑116^-/-^ and HT-29 cells.** A, miR‑215-5p significantly reduce the migration of HCT-116^-/-^ cells (scratch wound assay). B, miR‑215-5p significantly reduce the migration of HT-29^-^ cells (scratch wound assay). * *P* < 0.05, *** *P* < 0.001, CTRL = control cells

**
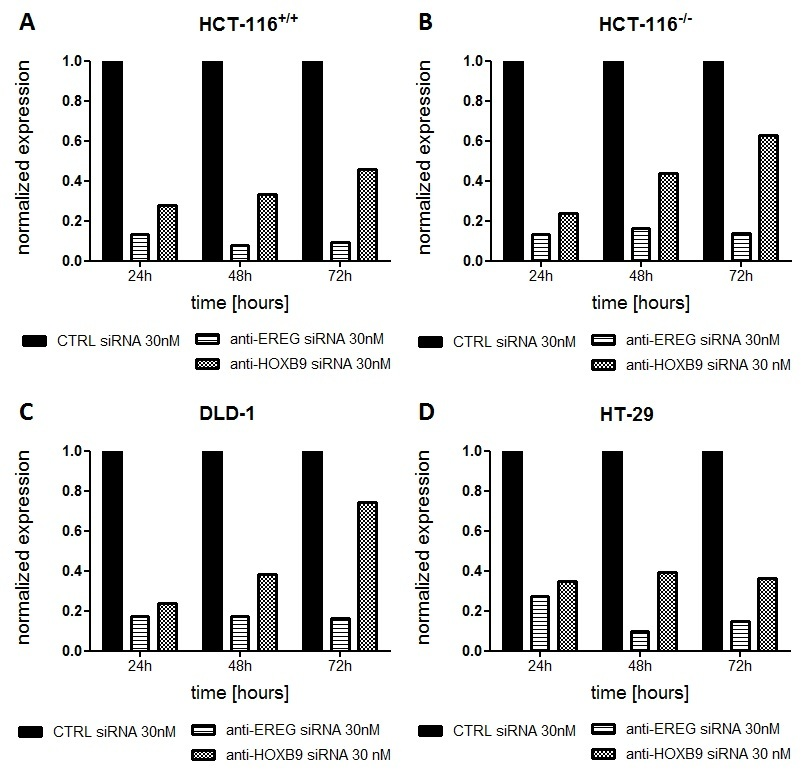
**

**Supplementary Figure S4 Inhibition of EREG and HOXB9 expression using small interfering RNAs.** A, Expression of EREG and HOXB9 was decreased in HCT-116^+/+^ cells 24-72 hours after transfection using anti-EREG and anti-HOXB9 siRNAs. B, Expression of EREG and HOXB9 was decreased in HCT‑116^-/-^ cells 24-72 hours after transfection using anti-EREG and anti‑HOXB9 siRNAs. C, Expression of EREG and HOXB9 was decreased in DLD-1 cells 24‑72 hours after transfection using anti-EREG and anti-HOXB9 siRNAs. D, Expression of EREG and HOXB9 was decreased in HT-29 cells 24-72 hours after transfection using anti-EREG and anti-HOXB9 siRNAs. CTRL = control cells

**
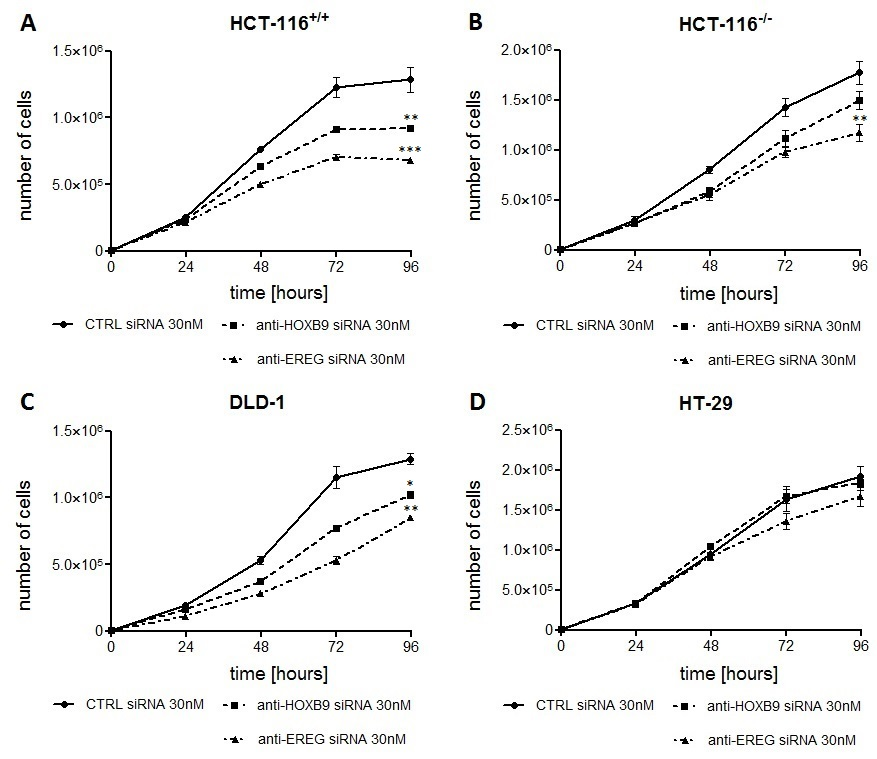
Supplementary Figure S5 Effect of EREG and HOXB9 inhibition on CRC cell proliferation.** A, Inhibition of EREG and HOXB9 lead to a significant decrease in HCT‑116^+/+^ cell proliferation. B, Inhibition of EREG and HOXB9 lead to a significant decrease in HCT-116^-/-^ cell proliferation. C, Inhibition of EREG and HOXB9 lead to a significant decrease in DLD-1 cell proliferation. D, Inhibition of EREG and HOXB9 did not significantly affect the proliferation of HT-29 cells. * *P* < 0.05, ** *P* < 0.01, *** *P*< 0.001, CTRL = control cells

**Supplementary Figure S6 Effects of miR-215-5p over-expression on E-cadherin (CDH1) levels.** miR-215**,** HCT-116^+/+^-miR-215-5p; NC, HCT-116^+/+^-control cells, * *P* < 0.05.

**Supplementary Figure S7 Expression of miR-215-5p in studied colorectal cancer cells.** A, calibration curve with synthetic miR-215-5p oligonucleotide. B, number of miR-215-5p copies per 100ng of RNA purified from studied CRC cancer cells.

**Supplementary Figure S8 Effects of miR-215-5p silencing on CaCo2 cells.** A, Efficacy of miR-215-5p silencing in CaCo2 cells. B, MiR-215-5p silencing induced increase in the expression of its targets EREG and HOXB9. C, MiR-215-5p silencing facilitated proliferation of CaCo2 cells, which was significant after two and three days of transfection. * *P* < 0.05
