# Supplementary figures and images for: MiR-215-5p is a tumor suppressor in colorectal cancer targeting EGFR ligand epiregulin and its transcriptional inducer HOXB9
Source: Oncogenesis. 2017 Dec 4;6(11):399. doi: 10.1038/s41389-017-0006-6 (PMC5868056; doi:10.1038/s41389-017-0006-6)

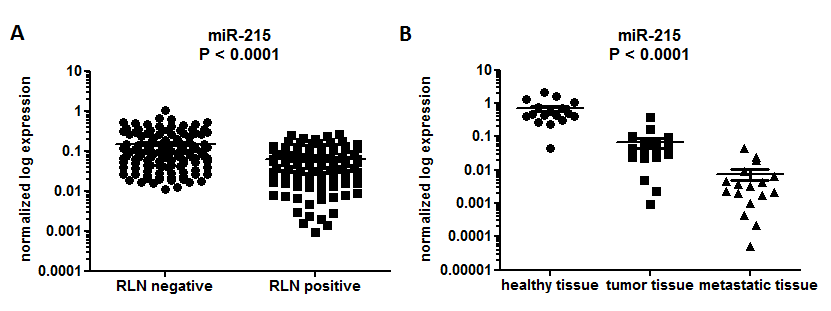

Supplement: Supplementary file 2 — Supplementary Figure S1 [file 41389_2017_6_MOESM2_ESM.tif]

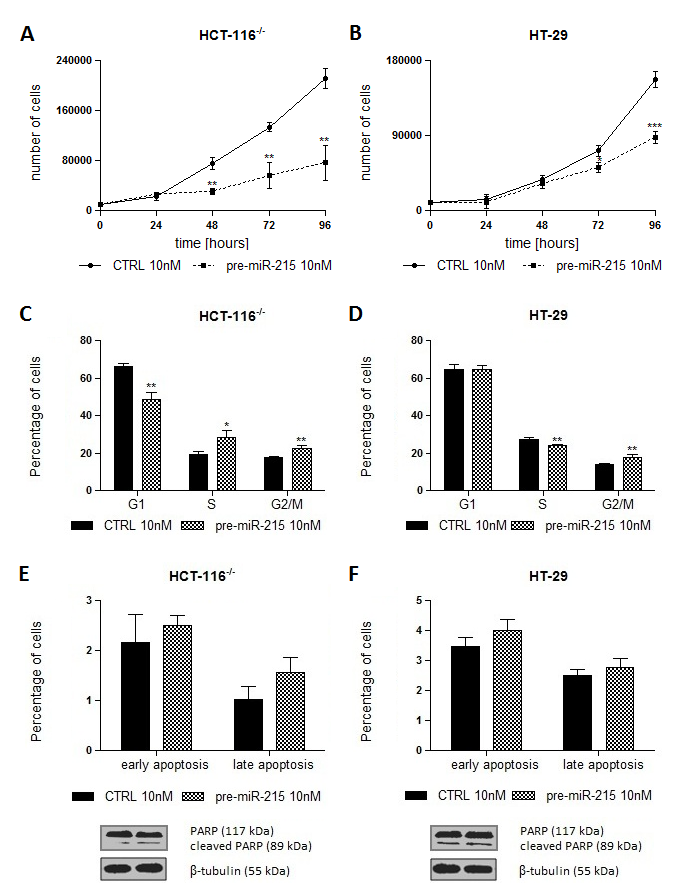

Supplement: Supplementary file 3 — Supplementary Figure S2 [file 41389_2017_6_MOESM3_ESM.tif]

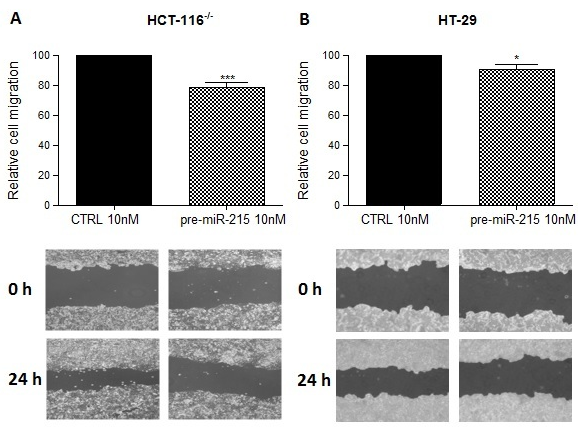

Supplement: Supplementary file 4 — Supplementary Figure S3 [file 41389_2017_6_MOESM4_ESM.tif]

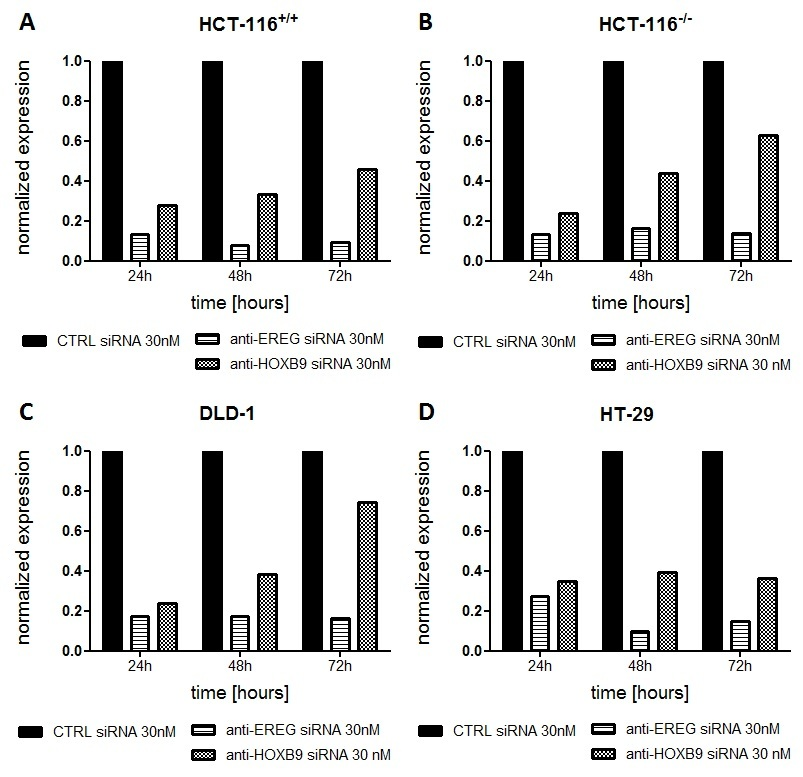

Supplement: Supplementary file 5 — Supplementary Figure S4 [file 41389_2017_6_MOESM5_ESM.tif]

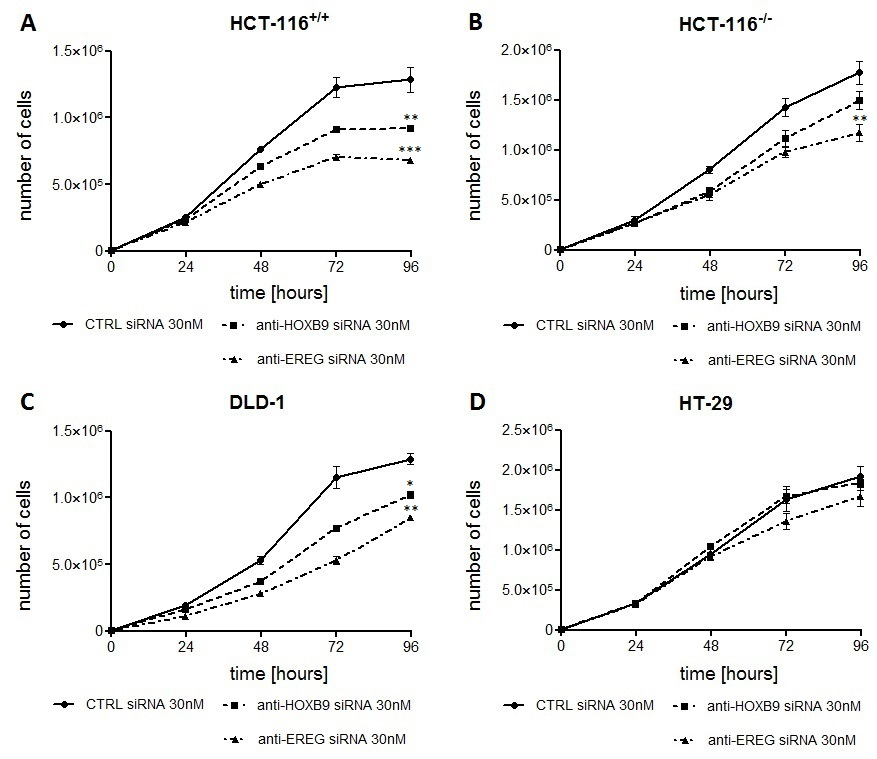

Supplement: Supplementary file 6 — Supplementary Figure S5 [file 41389_2017_6_MOESM6_ESM.tif]

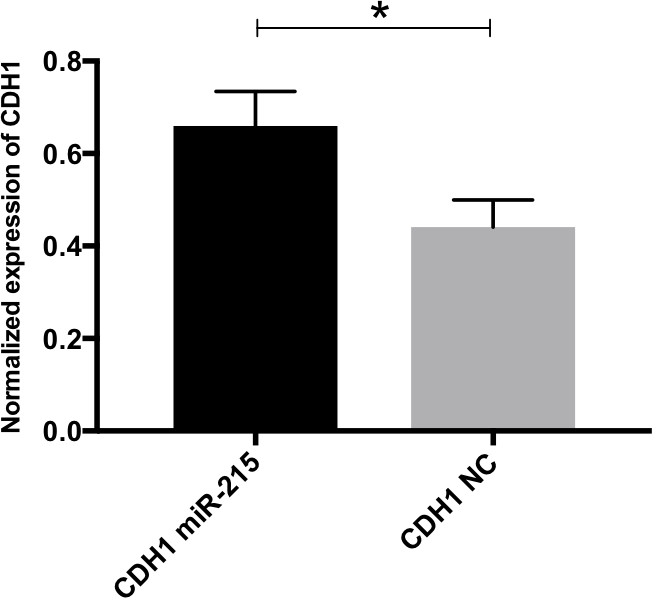

Supplement: Supplementary file 7 — Supplementary Figure S6 [file 41389_2017_6_MOESM7_ESM.tif]

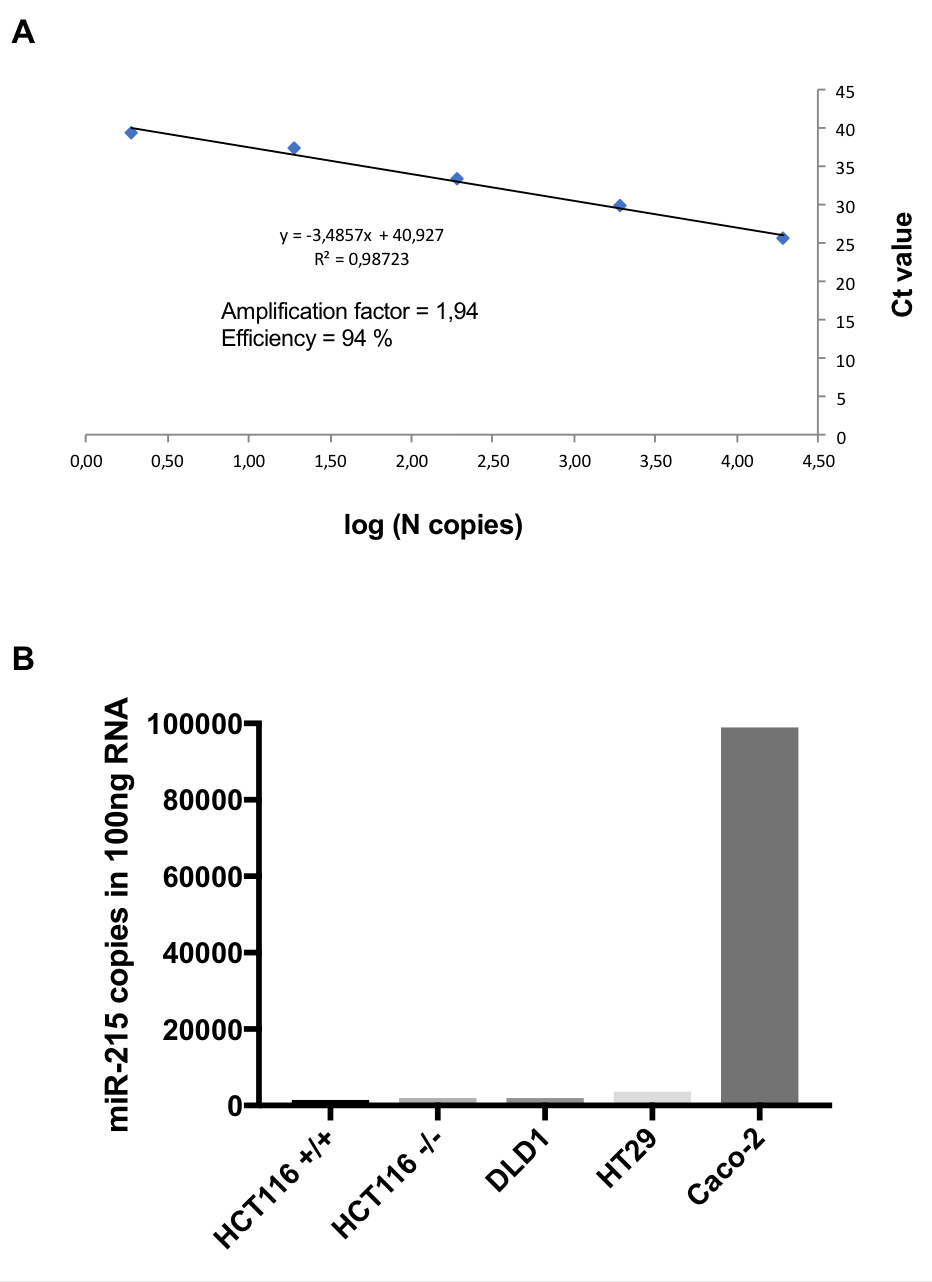

Supplement: Supplementary file 8 — Supplementary Figure S7 [file 41389_2017_6_MOESM8_ESM.tif]

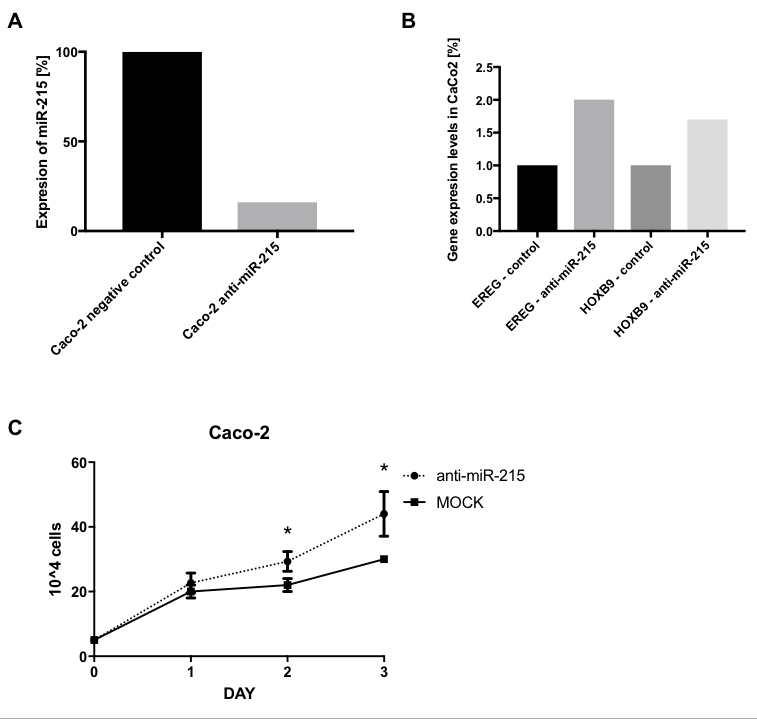

Supplement: Supplementary file 9 — Supplementary Figure S8 [file 41389_2017_6_MOESM9_ESM.tif]
